# Haptoglobin as a supplement in in vitro embryo culture: a tool for improving bovine embryo development and quality

**DOI:** 10.1186/s40659-025-00635-0

**Published:** 2025-08-20

**Authors:** Karina Cañón-Beltrán, Yulia N. Cajas, Cristina Martínez-López, Carla Moros-Nicolás, Pilar Sòria-Monzó, Miriam Balastegui-Alarcón, Manuel Avilés, Dimitrios Rizos, Francisco A. García-Vázquez, Mª José Izquierdo-Rico

**Affiliations:** 1 Department of Animal Reproduction, National Institute for Agriculture and Food Research and Technology (INIA-CSIC), Madrid, Spain; 2 Programa de Medicina Veterinaria y Zootecnia, Corporación Universitaria del Huila (CORHUILA), Grupo Kyron, Huila, Colombia; 3 Department of Biological Science, Technical University of Loja (UTPL), Loja, 1101608 Ecuador; 4 Departamento de Fisiología, Facultad de Veterinaria, Universidad de Murcia, Murcia, 30100 España; 5 Instituto Murciano de Investigación Biosanitaria Pascual Parrilla (IMIB), Murcia, España; 6 Campus de Excelencia Mare Nostrum (CMN), Murcia, España; 7 Departamento de Biología Celular e Histología, Facultad de Medicina, Campus de Ciencias de la Salud, Universidad de Murcia, Murcia, 30120 España

**Keywords:** Haptoglobin, Cattle, Embryo production, Uterus, Uterine fluid

## Abstract

**Background:**

Recent studies have indicated the potential involvement of haptoglobin in a variety of events during mammalian reproduction, with previous research highlighting its efficacy in promoting porcine embryo development. The present study aims to provide a comprehensive investigation of haptoglobin expression and secretion in the bovine uterus, with a view to assessing its impact on bovine in vitro embryo production. A systematic study was conducted on cows in different oestrous stages, early follicular, late follicular, early luteal and late luteal stages. Relative haptoglobin mRNA abundance was quantified by RT-qPCR, and the expression of the protein was analysed by immunohistochemistry. The results were complemented by proteomic analyses of the uterine fluid. In vitro bovine fertilisation and embryo culture were carried out in the presence of haptoglobin.

**Results:**

Haptoglobin mRNA expression in the bovine uterus is most abundant during the late luteal stage of the oestrous cycle. The presence of haptoglobin was demonstrated in the uterine epithelium and the uterine fluid in different stages of the oestrous cycle by immunohistochemistry and proteomic analyses. Furthermore, the addition of haptoglobin to in vitro culture improved the development of bovine embryos when the protein was present on the days corresponding to its passage through the uterus, with a higher blastocyst yield (*P* < 0.05) being observed in haptoglobin treatments compared with control groups. Haptoglobin appears to influence embryonic development by reducing mitochondrial activity and lipid content. Furthermore, transcripts associated with oxidative stress, lipid metabolism and cell cycle regulation were affected by the presence of haptoglobin.

**Conclusions:**

The presence of haptoglobin protein in the female tract of cows during different stages of the oestrous cycle suggests that it plays a significant role in the reproductive process. The addition of haptoglobin during in vitro embryo production resulted in enhanced blastocyst rates and improved quality.

**Supplementary Information:**

The online version contains supplementary material available at 10.1186/s40659-025-00635-0.

## Background

The secretions of the female genital tract are involved in important reproductive events, including the final maturation of gametes, fertilization, embryo development and implantation [1–4]. Before implantation, the embryo development depends on oviductal and uterine fluids (OF, UF), which are composed of plasma-derived components, and other substances synthesized by the epithelial cells and uterine glands, under the control of ovarian hormones [5–7].

The use of assisted reproductive technologies (ARTs) is widespread, not only in the field of animal production, but also in human medicine due to the significant rates of infertility. These technologies bypass the reproductive tract and use artificial media to produce embryos. However, the quality of in vitro-produced embryos is inferior to those produced in vivo, in terms of morphology, metabolism, epigenetic profile, cryotolerance and pregnancy rates [8–13]. Therefore, in recent years, attempts have been made to mimic the conditions of the physiological environment, for example, by adding proteins and other molecules to the in vitro fertilization (IVF) media or to the embryo culture (EC) media [14–16], or even the use of reproductive fluids as a culture media supplement [17–22].

In bovine species, the in vitro production of embryos (IVP) is a well-established technology. It is estimated that approximately 1.5 million embryos are transferred globally within this species (data from the International Embryo Technology Society, 2021). Nevertheless, the IVP system still needs to be improved, particularly in terms of embryo quality, given that the proportion of embryos that reach the transferable stage is approximately 30–40% [23]. A more complete understanding of the function of reproductive proteins could prove beneficial in the development of ARTs in a range of species.

Haptoglobin was first identified in 1939 [24]. This protein, which is synthesized in the liver, is known to bind free haemoglobin released from erythrocytes, inhibiting its oxidative activity. Furthermore, haptoglobin was traditionally considered an acute phase protein that operates within the physiological framework of an inflammatory response [25–27]. Haptoglobin is a common indicator of systemic inflammation in transition dairy cows, being associated with the degree of immune activation in the postpartum period [28, 29]. However, haptoglobin is now acknowledged as a typical constituent of healthy mammalian reproductive tissues and fluids, and haptoglobin expression has been demonstrated in the absence of inflammation. For example, in ovaries [30–32], oviducts [15, 32–34], uterus [15, 30, 34–39], testes [31], and in ovarian follicular fluid [32, 40–42], OF [15, 32, 34, 43, 44] and UF [15, 34, 45–48]. The presence of haptoglobin in reproductive fluids and tissues may indicate a role for this protein in reproduction. Indeed, previous experiments conducted by our research group have demonstrated that haptoglobin plays a crucial role in the early development of the porcine embryo. The presence of haptoglobin in the embryo culture media has been shown to increase blastocyst rate by 26.67% [15]. Therefore, haptoglobin may modulate the interaction of gametes or affect early embryo development. In light of the above, the objective of this study was to analyse the following aspects in cattle: (1) the relative abundance of mRNA encoding for the haptoglobin protein in the uterus in different phases of the oestrous cycle, (2) the presence of the protein in UF during the different phases of the oestrous cycle, (3) the localisation of the haptoglobin protein in uterus during the oestrous cycle, and (4) the haptoglobin function by means of functional analyses during IVP.

## Methods

### Reagents

Unless otherwise stated, all chemicals were purchased from Sigma-Aldrich Corporation (St Louis, MO, USA).

### Experimental design

**Fig. 1:**
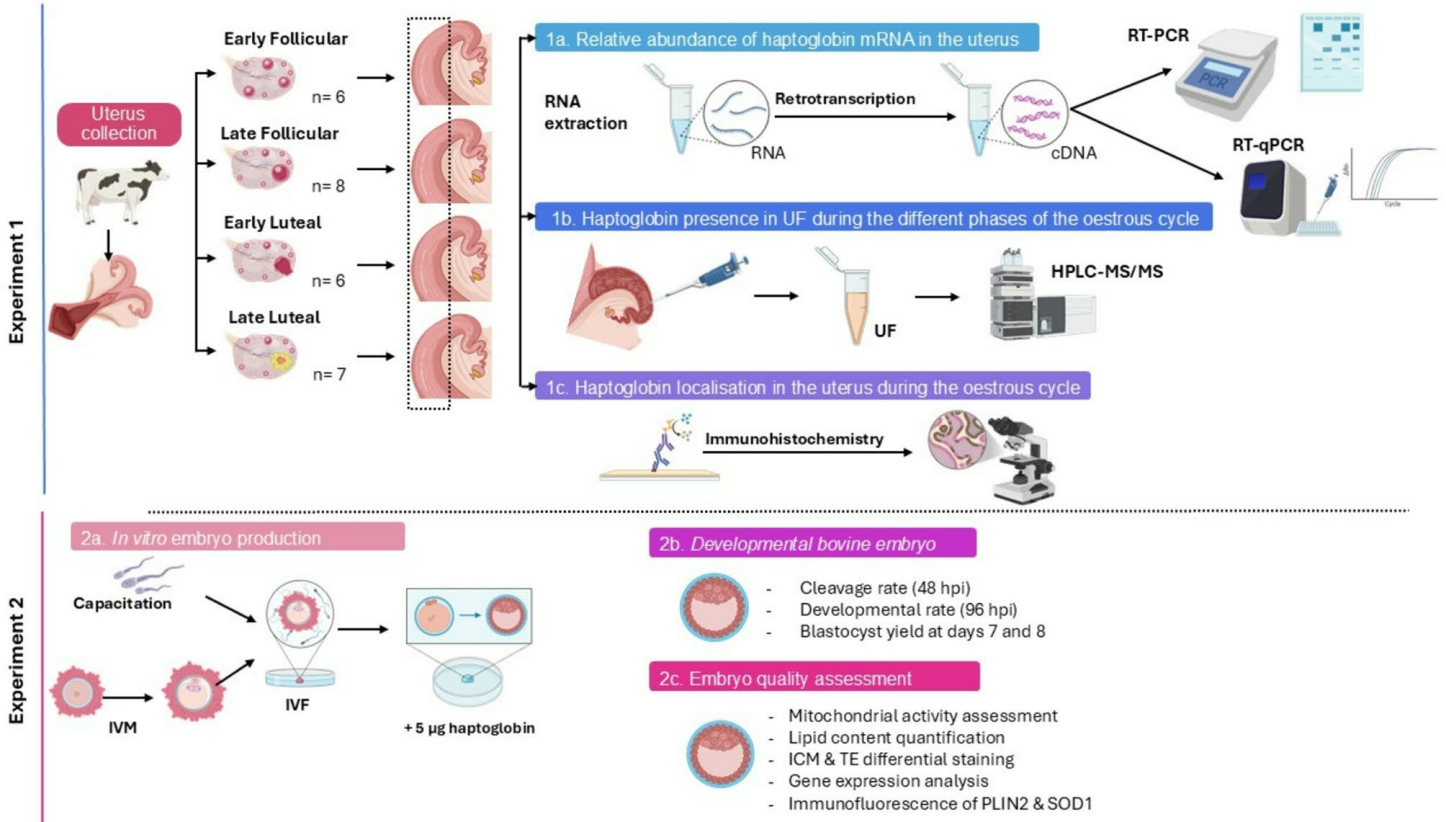
Experimental design with the aspects analysed in the present study: (**1a**) relative abundance of haptoglobin mRNA in the uterus, (**1b**) haptoglobin presence in UF during the different phases of the oestrus cycle, (**1c**) haptoglobin localisation in the uterus during the oestrous cycle, (**2a**) in vitro embryo production (**2b**) developmental bovine embryo, and (**2c**) embryo quality assessment

A schematic representation of experimental design is shown in Fig. 1. Female reproductive tracts from adult cows were obtained from a local abattoir (Mercamurcia, Murcia, Spain) within 15–20 min post-mortem. The stage of the oestrous cycle was determined based on ovarian morphology and classified as follows: (1) Early Follicular (EF), characterized by the presence of multiple small follicles; (2) Late Follicular (LF), characterized by a single dominant follicle; (3) Early Luteal (EL), marked by the presence of a haemorrhagic corpus luteum; and (4) Late Luteal (LL), defined by the presence of a mature corpus luteum.

#### Experiment 1. Analysis of uterine haptoglobin expression in cattle throughout the oestrus cycle

##### **Relative abundance of haptoglobin mRNA in the uterus**

Uterine tissue samples were obtained from the uterine horns by sectioning at the midpoint of the organ. The number of samples collected for each phase was as follows: EF (*n* = 6), LF (*n* = 8), EL (*n* = 6), and LL (*n* = 7). These tissues were used to perform reverse transcription polymerase chain reaction (RT-PCR) to detect haptoglobin expression in the uterus and reverse transcription quantitative real-time polymerase chain reaction (RT-qPCR) to quantify gene expression levels across the different phases of the oestrous cycle.

##### **Haptoglobin presence in UF during the different phases of the oestrus cycle**

UF was collected from cows at each oestrous phase: EF (*n* = 6), LF (*n* = 8), EL (*n* = 6), and LL (*n* = 7) and pooled. Samples were analysed by tandem mass spectrometry (MS/MS) to identify the presence of haptoglobin protein.

##### **Haptoglobin localisation in the uterus during the oestrous cycle**

Uterine tissue sections (*n* = 3 per phase) were processed for immunohistochemical analysis to localize haptoglobin expression within the uterine tissue.

#### Experiment 2. In vitro effect of haptoglobin supplementation on bovine embryo development and quality

##### **Preliminary experiment: establishment of optimal haptoglobin concentration on early embryo development in vitro**

The optimal concentration of haptoglobin in IVC media for early embryo development, under our experimental conditions, was established based on findings from previous studies [15, 34]. In this preliminary experiment, presumptive zygotes from four individual replicates were cultured in IVC medium alone (Control; *n* = 448) or supplemented with haptoglobin (H) (Bovine haptoglobin, Alpha Diagnostic International) at 20 µg/mL (H20; *n* = 126), 10 µg/mL (H10; *n* = 292) and 5 µg/mL (H5; *n* = 348) until day 8. The developmental parameters were calculated as follows: (I) cleavage rate at 48 hpi: percentage of presumptive zygotes that developed to the 2-cell stage; (II) developmental rate at 96 hpi: percentage of embryos that developed to the 16-cell stage; and (III) blastocyst yield: percentage of embryos that developed to the blastocyst stage at days 7 and 8.

##### **Effect of haptoglobin supplementation on the developmental bovine embryos**

The present experiment sought to investigate the effect of the addition of haptoglobin (5 µg/mL) to the IVC media on the development and quality of bovine embryos during two distinct developmental periods. For this experiment, presumptive zygotes were cultured in IVC medium alone (Control; *n* = 516) or supplemented with 5 µg/mL of haptoglobin (H5; *n* = 519) during the entire culture period (day 1 to day 8) or during two developmental period: day 1 to day 4: from presumptive zygotes to 16-cell stage (representing haptoglobin effect in the oviduct; H5-C: *n* = 446); or day 4 to day 8: from 16-cell to blastocyst stage (representing haptoglobin effect in the uterus; C-H5: *n* = 438), maintaining the different experimental groups separately.

The developmental parameters evaluated were: (I) cleavage rate at 48 hpi; (II) developmental rate at 96 hpi; and (III) blastocyst yield at days 7 and 8. Ten replicates were performed under the same assay conditions.

##### **Effect of haptoglobin supplementation on the of quality bovine embryos**

To evaluate whether haptoglobin influences the quality of bovine embryos, mitochondrial activity, lipid content and expression levels of genes related to embryo development and quality were evaluated.

For mitochondrial activity and lipid content analyses, 16-cell embryos (*n* = 15) and blastocysts (D7) (*n* = 20) from each treatment (Control, H5, H5-C and C-H5) were stained with MitoTracker DeepRed and Bodipy 493/503, respectively. For gene expression analysis, a representative number of embryos at the 16-cell and D7 blastocyst stages were collected from each experimental group (Control, H5, H5-C, and C-H5). For each stage and group, three independent pools of 10 embryos (*n* = 30 embryos per group/stage) were prepared, snap-frozen in LN_2_, and stored at -80 °C until analysis. The selected genes (POU Domain, Class 5, Transcription Factor 1 (POU5F1), Cyclin-dependent Kinase 2 (CDK2), Chromodomain Helicase DNA Binding Protein 1 (CHD1), PPARG Coactivator 1 Beta (PPARGC1B), Superoxide Dismutase 1 (SOD1), Perilipin 2 (PLIN2), Glutathione Peroxidase 1 (GPX1), Nuclear Factor Erythroid 2-Related Factor 2 (NFE2L2), and BCL2 associated X, apoptosis regulator (BAX)) have been linked to embryonic development and metabolic pathways and are essential in cell proliferation, differentiation and embryo quality [49–53].

To determine whether haptoglobin modulates embryo development by affecting the blastocyst cell number (TE and ICM), a representative number of blastocysts (D7) from each group (20 per group) was incubated with an anti-CDX2 antibody and then stained with Hoechst 33342 (10 µg/mL). Blastocysts were examined under a confocal microscope, and the obtained images were evaluated using the ImageJ software (National Institutes of Health (NIH), Bethesda, MD, USA, version 1.52 k software (https://imagej.net/ij/). Additionally, blastocysts (D7) (15 per group) from all experimental groups were fixed in 4% PF for immunofluorescence analysis of PLIN2 and SOD1, which are associated with lipid metabolism and oxidative stress, respectively.

### RNA isolation and cDNA synthesis

Uterus samples were collected and stored in RNAlater in a -80 °C freezer until use. RNA extraction was performed using the RNAqueous™ Phenol-free Total RNA Isolation Kit (Invitrogen, Carlsbad, CA, USA) according to the manufacturer’s instructions. The reverse transcription reaction was performed using the QuantiTect Reverse Transcription Kit (Qiagen, Hilden, Germany).

### Reverse transcription polymerase chain reaction

Bovine haptoglobin cDNA was amplified by RT-PCR (from uterine horn and body), specific primers were designed with the NCBI primer tool according to the predicted cDNA sequences in the GenBank database with accession number NM_001040470. β-actin (ACTB; Accession no. NM_173979) was used as a positive control. The primers were validated using oligonucleotides properties calculator (https://www.sigmaaldrich.com/pc/ui/easy-oligo-home/easyoligo) and BLAST tool (https://blast.ncbi.nlm.nih.gov/Blast.cgi) (Additional file 1). RT-PCR was performed using the KAPA2G Fast HotStart ReadyMix (Roche, Basel, Switzerland) in a Bibby Scientific Prime Thermal Cycler (Thermo Fisher Scientific) with an initial denaturation cycle of 3 min at 95 °C, followed by 35 cycles of 15 s at 95 °C, 15 s at 55 °C, and then 1 s at 72 °C. The final extension time was 1 min at 72 °C. The generated amplicons were visualized by 1.5% agarose gel electrophoresis and purified using the FavorPrep™ GEL/PCR Purification Kit (Favorgen Biotech Corp., Vienna, Austria) to be sequenced by Sanger using a 3500 GeneticAnalyzer (Applied Biosystems).

### Reverse transcription quantitative real-time polymerase chain reaction

The relative expression levels of haptoglobin mRNA in bovine uterus at various phases of the oestrous cycle, including EF, LF, EL, and LL phases, were determined using bovine haptoglobin-specific SYBR Green primers designed for haptoglobin, and ACTB obtained from Sigma Aldrich (Additional file 1). RT-qPCR was performed by the Genomic Platform at the IMIB-Arrixaca using SYBR Premix Ex Taq II (Tli RNaseH Plus, Takara) in the QuantStudio 5 Real-Time PCR System (Applied Biosystems). Each PCR reaction was conducted in a 5 µl volume with a primer concentration of 450 nM. The cycling conditions consisted of an initial denaturation step at 95 °C for 30 s, followed by 40 cycles of denaturation at 95 °C for 5 s and annealing/extension at 60 °C for 34 s. All samples were run in triplicate. Gene expression analysis was performed using the 2^-ΔΔCt method, where ΔCt represents the difference between the threshold cycle of a given target cDNA and ACTB gene.

### Collection of uterine fluid

UF from cows at different oestrous phases were collected and pooled. Briefly, uterine horns were dissected, UF was collected by applying pressure with a tip (horizontally positioned) from the uterine body to the end of the horns and aspirated with an automatic pipette. The fluid was centrifuged twice at 800 g for 5 min at 4 °C to remove debris before aliquoting and storing at -20 °C until use.

### HPLC–MS/MS analysis

#### In-gel Trypsin digestion

Samples were digested with the following standard procedure. After electrophoresis and image analysis, selected bands were spliced in approximately 2 × 2 mm parts and destained. Then, bands were reduced with 25 mM ammonium bicarbonate buffer pH 8.5 with 10 mM DTT followed by alkylation with 25 mM ammonium bicarbonate buffer pH 8.5 with 25 mM iodoacetamide. After washing, samples were digested with 25 mM ammonium bicarbonate buffer pH 8.5 containing 0.5 µg of Trypsin Gold Proteomics Grade (Promega Corporation, Madison, MI, USA) and 0.01% ProteaseMax surfactant (Promega Corporation, Madison, MI, USA). Finally, samples were dried using the vacuum evaporator.

#### HPLC-MS/MS analysis

The separation and analysis of the tryptic digests of the samples were performed as previously described [54] with a HPLC/MS system consisting of an Agilent 1290 Infinity II Series HPLC (Agilent Technologies, Santa Clara, CA, USA) equipped with an Automated Multisampler module and a High Speed BinaryPump, and connected to an Agilent 6550 Q-TOF Mass Spectrometer (Agilent Technologies, Santa Clara, CA, USA) using an Agilent Jet Stream Dual electrospray (AJS-Dual ESI) interface.

### Immunohistochemistry

Uterus samples were formalin-fixed, paraffin embedded and sectioned (4 μm-thick). To assess the immunolocalization of haptoglobin, an indirect immunohistochemical procedure was carried out. After deparaffination and rehydration, the sections were undergone to a demasking a heat-induced antigen procedure (PT link, Thermo Scientific, Madrid, Spain) by using a commercial solution (target retrieval solution, low pH. GV-805. Agilent-Dako, Barcelona, Spain). After endogenous peroxidase blocking, sections were incubated for 20 min at 37 °C with normal horse serum (30022, Vector Labs., Burlingame, CA) to block endogenous background and then 4 °C overnight with the primary antibody (rabbit anti-human haptoglobin polyclonal antibody, Creative Diagnostics, NY 11967, USA) (dilution 1:1000). To amplify the immunohistochemical signal, the sections were firstly incubated for 15 min at 37 °C with a rabbit linker commercial solution (EnVision Flex, Rabbit Linker, Agilent-Dako) and then with the HRP-labelled anti-rabbit polymer system (ImmPress anti-rabbit. 30026. Vector Labs) for 30 min at 37 °C. The immunoreactivity was finally revealed with a 3–3´diaminobencidine commercial kit (GV825, Dako-Agilent) and contrasted with Harry´s hematoxylin (Thermo Scientific), dehydrated, cleared and mounted.

For microscopic examination, the sections were digitized with a high-resolution brightfield slide scanner (Pannoramic MIDI II, 3D Histech, Budapest, Hungary) and digital visualized by using a specific software (Slide Viewer, Ver. 2.6.0.166179, 3D Histech). Representative images were also obtained by using the same software.

### In vitro embryo production

A commercially produced un-defined media set (Bovine oocyte in vitro maturation (IVM), IVF and in vitro culture (IVC) media, Wash Media (WM), Semen Wash (SWM) and Stroebech Heavy-Oil) (Stroebech Media, Hundested, Denmark) was used for embryo production. All media were prepared according to the manufacturer’s recommendations and pre-warmed at 38.5 °C before use, unless indicated otherwise.

Immature cumulus-oocyte complexes (COCs) were obtained by aspirating follicles (2–8 mm) from the ovaries of mature heifers and cows obtained from local abattoirs. The collected COCs were washed three times in WM. Then, the COCs were placed into 4-well dishes (Nunc, Roskilde, Denmark) containing 500 µL of IVM medium, with 50 COCs per well. The COCs were incubated for 24 h at 38.5 °C and under an atmosphere of 5% CO_2_ in saturated humidity.

### Sperm preparation and in vitro fertilization

Matured oocytes were transferred to the IVF medium and kept in the incubator until the semen was prepared. Frozen semen straws (0.25 mL) from a previously IVF-tested Asturian Valley bull were thawed at 37 °C in a water bath for 1 min. The thawed semen was transferred to a centrifuge tube containing 4 mL of SWM and centrifuged at 300 g for 5 min. After centrifugation, the supernatant was removed, and 3 mL of SWM was added to the tube. The sample was centrifuged again at 300 g for 5 min, and the supernatant was removed. Finally, sperm concentration was determined and adjusted to a final concentration of 2 × 10^6^ sperm cells/ mL. Gametes were co-incubated for 18–22 h in 500 µL of IVF medium in a four-well dish, with 50 COCs per well, under an atmosphere of 5% CO_2_ with maximum humidity at 38.5 °C. The sperm motility was evaluated by direct observation under light microscopy during the co-incubation process. The estimated motility of the sperm suspension was approximately (70–80%).

### In vitro embryo culture

At 18–22 h post-insemination (hpi), presumptive zygotes were transferred into a 15 mL tube with 2 mL WM and vortexed at a fast speed for 2 min. After this procedure, 20–25 presumptive zygotes were cultured in 25 µL droplets of IVC covered with Stroebech Heavy-Oil. Culture conditions were as follow: 38.5 °C and an atmosphere of 5% CO_2_, 5% O_2_ and 90% N_2_. Cleavage rate was analysed on day 2 (48 hpi) and developmental rate was recorded at 96 hpi. Additionally, cumulative blastocyst yield was recorded on days 7 and 8 post-insemination (dpi) under a stereomicroscope.

### Embryo quality evaluation by mitochondrial activity measurement and lipid content quantification

For mitochondrial activity, 16-cell embryos and blastocysts were suspended in 100 µL phosphate-buffered saline (PBS) without calcium and magnesium supplemented with 0.1% polyvinylpyrrolidone (PVP). Then, embryos were equilibrated for 15 min in culture medium supplemented with 5% fetal calf serum (FCS) and then incubated for 30 min at 38.5 °C in 400 nmol/mL MitoTracker DeepRed (Molecular Probes, Eugene, USA). Finally, samples were then fixed in 4% paraformaldehyde (PF) for 30 min at room temperature.

For lipid content analysis, 16-cell embryos and blastocysts were fixed in PF and then permeabilized with 0.1% saponin for 30 min and stained for 1 h with 20 µg/mL Bodipy 493/503. For total cell number count, the blastocysts were stained with Hoechst 33,342 (10 µg/mL) for 30 min, after being stained either for mitochondria or for lipids. After each stain, the samples were washed in PBS + 0.1% PVP three times for 5 min each. Finally, samples were mounted in 3.8 µL mounting medium (ProLong Gold, Thermo Fisher Scientific) between a coverslip and a glass slide and sealed with nail polish. Slides were examined using a laser-scanning confocal microscope (Leica TCS SP2) equipped with an argon laser excited at 488 nm and whose emission spectrum is 500–537 nm for visualization of lipid droplets. For mitochondria, excitation and emission were set at 543 nm and 580–650 nm, respectively. The format, laser, gain and offset were kept constant for every sample.

For the assessment of mitochondrial activity, the fluorescence signal intensity (pixels) was quantified. Serial sections of 5 μm were made for each sample and a maximum projection was accomplished for each one. Images obtained were evaluated using the ImageJ program. After selection using the freehand selection tool, each sample was measured to determine its area and its integrated density (IntDen), which corresponds to pixel intensity. In addition, the background fluorescence of an area outside the embryos was measured. Fluorescence intensity in each sample was determined using the following formula: Relative fluorescence = IntDen – (area of selected embryo/blastocyst × mean fluorescence of background readings). Fluorescence intensities are expressed in arbitrary units (a.u.) [19, 49].

The lipid quantity was obtained by analysing the total area of lipids in each embryo. A 63× objective at a resolution of 1024 × 1024 was used and images were analysed using the ‘nucleus counter’ tool, set to detect, distinguish, and quantify droplet areas with the ImageJ software and expressed as the area of lipid droplets relative to the total area of the blastocyst without its cavity (µm^2^). The number of cells per blastocyst was determined by counting the Hoechst-stained cells under an epifluorescence microscope (Nikon 141,731) equipped with a fluorescent lamp (Nikon HB-10104AF) and UV-1 filter.

### Embryo quality evaluation by differential staining of blastocysts

Differential staining of inner cell mass (ICM) and trophectoderm (TE) cells was carried following the procedures of Cajas et al. (2021) [55]. The zona pellucida of blastocysts (~ 20 per group) was removed with 0.5% (w/v) pronase in PBS. Zona-free embryos were washed in PBS three times and were then fixed in 4% PF in PBS supplemented with 1% bovine serum albumin (BSA) (PBS + 1% BSA) for 10 min at room temperature. After fixation, embryos were washed three times in PBS + 1% BSA and kept in that medium at 4 °C until analysis. For immunostaining, cells were permeabilized in PBS + 5% goat serum (IF buffer) with 1% Triton X-100 for 45 min at room temperature. Blastocysts were then incubated overnight at 4 °C in primary antibody solution consisting of PBS + 1% BSA, 20% IF buffer and 1:1000 mouse monoclonal anti-CDX2 antibody (Biogenix, Fremont, CA, USA). Following incubation, blastocysts were washed twice in PBS + 1% BSA and incubated in the secondary antibody solution consisting of PBS + 1% BSA, 20% IF buffer, 1:3000 Alexa Fluor goat anti mouse 488 (Invitrogen, Carlsbad, CA, USA) and Hoechst 33,342 (10 µg/mL) for 2 h at room temperature. Finally, embryos were washed three times in PBS + 1% BSA and mounted in 3.8 µL of mounting medium between a coverslip and a glass slide and sealed with nail polish. Stained blastocysts were analyzed by a confocal microscope (Carl Zeiss Microscopy GmbH, Jena, Germany). Z-stack sections of 5 μm were taken in the 405 nm (Hoechst positive cells, total cell number) and 488 nm (CDX2-positive cells, trophectoderm cells) channels. Images obtained were evaluated using the ImageJ program.

### Gene expression analysis of blastocysts

For gene expression analysis, blastocysts were washed in PBS, snap-frozen in LN_2_ and stored in a -80 °C freezer until mRNA extraction analyses. Poly(A) RNA was extracted using the Dynabeads mRNA Direct Extraction Kit (Ambion; Thermo Fisher Scientific) with minor modifications [50, 55]. Immediately after poly(A) RNA extraction, reverse transcription (RT) was performed using a MMLV Reverse Transcriptase 1st-Strand cDNA Synthesis Kit according to the manufacturer’s instructions (Epicentre Technologies). Poly(T) random primers and Moloney murine leukaemia virus (MMLV) high-performance reverse transcriptase enzyme were used in a total volume of 40 µL to prime the RT reaction and to produce cDNA. Tubes were heated to 70 °C for 5 min to denature the secondary RNA structure and the RT mix was then completed by adding 50 units of reverse transcriptase. Samples were incubated at 25 °C for 10 min, to help the annealing of random primers, followed by incubation at 37 °C for 60 min, to allow the RT of RNA, and finally at 85 °C for 5 min to denature the enzyme. All mRNA transcripts were quantified in duplicate using a Rotorgene 6000 Real Time Cycler (Corbett Research). RT-qPCR was performed by adding 2 µL aliquot of each cDNA sample (~ 60 ng/µL) to the PCR mix (GoTaq qPCR Master Mix, Promega) containing the specific primers to amplify transcripts for the genes (Additional file 2). All primers were designed using Primer-BLAST software (http://www.ncbi.nlm.nih.gov/tools/ primer- blast/) to span exon–exon boundaries when possible. For quantification, RT-qPCR was performed as described previously [50, 55]. The PCR conditions were tested to achieve efficiencies close to 1. Relative expression levels were quantified by the comparative cycle threshold (Ct) method [56]. Values were normalized using two housekeeping (HK) genes: H2AZ1 and ACTB. Fluorescence was acquired in each cycle to determine the threshold cycle or the cycle during the log-linear phase of the reaction at which fluorescence increased above the background for each sample. Within this region of the amplification curve, a difference of one cycle is equivalent to a doubling of the amplified PCR product. According to the comparative Ct method, the ΔCt value was determined by subtracting the mean Ct value of the two HK genes from the Ct value of the gene of interest in the same sample. The calculation of ΔΔCt involved using the highest treatment ΔCt value (i.e. the treatment with the lowest target expression) as an arbitrary constant to subtract from all other ΔCt sample values. Fold-changes in the relative gene expression of the target were determined using the formula 2^−ΔΔCt^.

### Immunofluorescence of PLIN2 and SOD1 in blastocysts

Immunolocalization of PLIN2 and SOD1 was performed according to Cajas et al. (2021) [55] with minor modifications. For this analysis, blastocysts were washed twice with PBS + 0.1% PVP and fixed in 4% PF for 10 min at room temperature. Next, embryos were permeabilized by incubation in PBS with 10% FCS and 1% Triton X-100 for 45 min at room temperature. After permeabilization, the samples were incubated overnight at 4 °C in PBS + 0.1% PVP and 5% FCS and 1:100 SOD1 mouse monoclonal antibody 8B10 (ThermoFisher, Berkeley, MO, USA, Cat # MA1-105) or 1:100 mouse monoclonal PLIN2 antibody (also known as ADRP, SC32450 Santa Cruz Biotechnology, Inc., Dallas, TX, USA). Following incubation, the samples were washed twice in PBS + 0.1% PVP and incubated in PBS supplemented with 5% FCS and 1:250 PBS supplemented with 5% FCS and 1:250 Alexa Fluor goat anti-mouse 488 (Invitrogen, Carlsbad, CA, USA), for 2 h at room temperature followed by washing again three times in PBS + 0.1% PVP. In all cases, nuclei were stained with Hoechst 33,342 (10 µg/mL). Finally, the samples were mounted in microdrops with Fluoromount G (EMS, Hatfield, UK) and examined by confocal microscopy (Leica TCS-SPE). Negative control was prepared omitting the primary antibody before adding the secondary antibody.

### Statistical analysis

The Ct values obtained from the RT-qPCR were analysed using the statistical software Thermo Fisher Connect Platform. Data normalization was performed using the ACTB gene as an endogenous control, and the early follicular phase as the reference group to calculate fold changes. Statistical differences between groups were evaluated using SPSS software (IBM SPSS Statistics^®^ software.

version 28.0.0 (IBM Corp., Armonk, NY, USA)). Normality was tested with the Shapiro-Wilk test, and homogeneity of variances was assessed using the Levene’s test. Since variances were not homogeneous, a non-parametric Kruskal-Wallis test was applied. Differences were considered statistically significant when *p* < 0.05.

For IVP data were analysed using SigmaStat (Jandel Scientific, San Rafael, CA, USA). Mitochondrial activity and lipid content, cleavage and blastocysts rates and relative mRNA abundance were normally distributed with homogeneous variance, so one-way analysis of variance (ANOVA), followed by Tukey´s test, were performed to evaluate the significance of differences between groups. Values were considered significantly different at *P* < 0.05. Unless otherwise indicated, data are presented as the mean ± SEM.

## Results

### Transcript expression analysis of bovine haptoglobin in the uterus across oestrous cycle phases

**Fig. 2:**
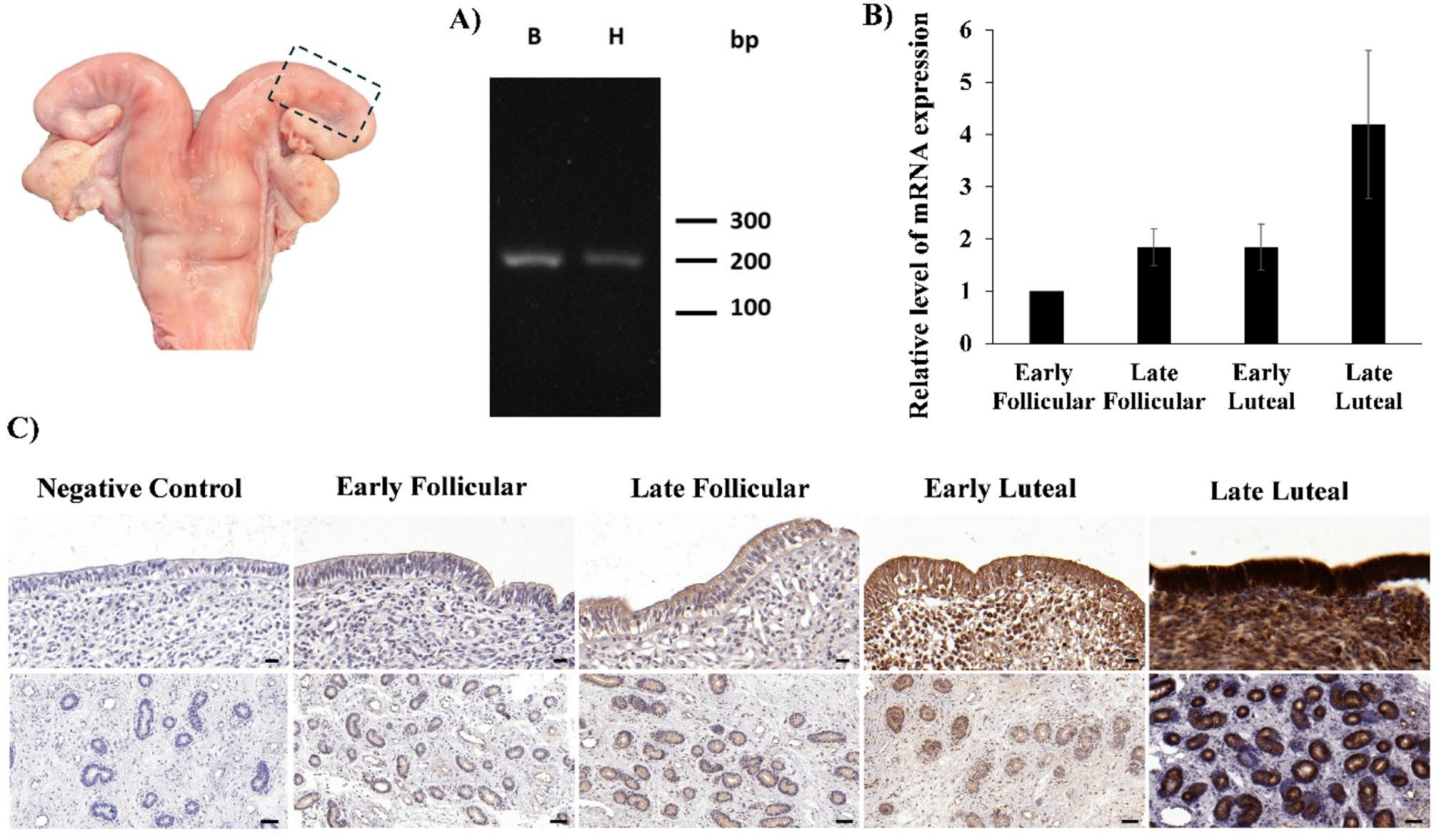
Expression of haptoglobin in the bovine uterus across different oestrous cycle phases. **(A)** Analysis of haptoglobin gene expression in the bovine uterus (body (B) and horn (H)) as determined by reverse transcription polymerase chain reaction (RT-PCR). A 200 bp amplicon is shown, confirming the expression of haptoglobin. **(B)** Relative mRNA expression levels of haptoglobin in the bovine endometrium throughout the oestrous cycle, as determined by reverse transcription quantitative real-time polymerase chain reaction (RT-qPCR). Expression levels are normalized to the early follicular phase (set as 1). Data are presented as mean ± standard error. **(C)** Immunohistochemical analysis of endometrial sections from bovine uterus at different oestrous cycle phases. The top row shows the distribution of haptoglobin in the luminal epithelium and lamina propria (scale bar = 20 μm). The bottom row shows the distribution of haptoglobin in the endometrial glandular structures (scale bar = 50 μm). A negative control (without primary antibody) was included to confirm staining specificity

In this study the cDNA of bovine haptoglobin in the uterus (body and horn) at EF phase was partially amplified by RT-PCR (Fig. 2A). The sequencing of an amplicon of 200 bp demonstrated that it exhibited 100% identity with the GenBank cDNA sequence with accession number KR006344.

The expression levels of haptoglobin across different phases of the bovine oestrous cycle were determined by RT-qPCR. The study revealed differential expression of haptoglobin during the oestrous cycle. The lowest expression was observed during the EF phase, while the highest expression was detected in the LL phase. Intermediate expressions were recorded in the EL and LF phases (Fig. 2B).

### Immunodetection of haptoglobin in the uterus

The present study evaluated the distribution of haptoglobin in the uterus using immunohistochemistry in order to ascertain its localization and potential role in reproductive events. Haptoglobin signal was localized predominantly in luminal epithelial cells and glandular cells, with a stronger signal in luteal phase than follicular phase. No signal was observed in the control (Fig. 2C).

### Proteomic detection of haptoglobin in uterine fluid across oestrous cycle phases

In this study, a proteomic analysis was performed to detect haptoglobin in UF samples in the different stages of oestrus cycle (EF, LF, EL and LL). The HPLC-MS/MS analysis detected a total of 52 different haptoglobin peptides in UF, giving a sequence coverage of 67.83% with respect to the primary sequence as derived from the gene (NP_001035560) (Table 1 and Additional file 3). When analysed by the stage of the oestrus cycle, 12 peptides were detected in the EF phase, corresponding to a sequence coverage of 29.18%, in the LF phase 24 peptides were identified (40.4% coverage), in the EL phase 27 peptides were identified (55.11% coverage) and in the LL phase 35 peptides were identified (56.36% coverage). Considering that HPLC-MS analysis can be considered as a semiquantitative technique, the higher sequence coverage observed during the luteal phases likely reflects a greater abundance of haptoglobin during these phases of the oestrus cycle.

**Table 1:** Peptides corresponding to haptoglobin protein detected by HPLC-ESI-MS/MS. When the same peptide was detected several times, the data corresponding to the peptide with the highest score is shown. Sequence: order of appearance of amino acids in the protein. Z: charge of the detected ion or fragment. M/z: mass/charge ratio. Score: score based on signal strength. SPI: scored peak intensity. Sample: stage of the cycle when the peptide was detected: early follicular (EF), late follicular (LF), early luteal (EL), and late luteal (LL)

Peptides	Sequence	z	m/z	Number of detections	Score	SPI	Sample
LQAVVTLLL	4–12	2	525.3148	1	5.32	72.8	LL
VVTLLLCGQLLAVETG	7–22	3	596.9457	1	5.56	62.4	LL
VVTLLLCGQLLAVETGSE	7–24	3	634.6573	4	9.52	65.2	EF, LF, EL, LL
VTLLLCGQLLAVETGSEAT	8–26	3	639.9887	1	5.60	65.5	EL
VTLLLCGQLLAVETGSEATA	8–27	3	682.6826	2	5.71	62.0	EF, EL
LLLCGQLLAVET	10–21	3	451.5763	1	5.34	67.3	EL
LLLCGQLLAVETGSEA	10–25	3	566.2834	2	6.68	67.4	EF, LF
LLCGQLLAV	11–19	2	493.7716	1	8.91	75.6	LF
LLCGQLLAVE	11–20	2	558.3141	1	6.56	63.8	EL
LLCGQLLAVETGS	11–23	2	680.8778	5	6.28	63.6	LF, EL, LL
LLCGQLLAVETGSEATADSCPKAP	11–34	4	622.8037	1	6.05	60.3	LF
QLLAVETGSEATADSCPKAPE	15–35	3	759.6612	4	7.05	67.2	EF, LF, EL, LL
VYTFNNKQWINKDIGQQLP	61–79	3	822.7101	1	6.89	66.5	EL
WINKNIGQKLPECEAV	128–143	2	950.0122	3	9.09	66.5	LF, EL, LL
INKNIGQK	129–136	2	457.7732	1	6.11	70.0	LF
AVCGKPKHPVDQVQRIIGGSLDAKGSFPWQA	142–172	4	837.4299	1	7.74	66.8	EL
HPVDQVQRIIG	149–159	2	631.3431	1	6.25	72.0	LL
IIGGSLDAK	157–165	2	477.2386	5	7.48	71.3	EF, LF, EL, LL
IIGGSLDAKGS	157–167	2	509.3005	3	6.89	66.5	LF, EL, LL
IGGSLDAK	158–165	2	380.7238	1	7.68	64.8	LL
IGGSLDAKGSFPWQAK	158–173	3	581.2896	1	9.94	70.1	EF
LISGATLINE	180–189	2	555.7895	2	6.32	66.6	LF, LL
ISGATLINERWLLTTA	181–196	3	613.6578	2	5.35	66.4	EF, LL
SGATLINERWLLTTAKNLYLGHSS	182–205	3	909.1159	1	8.50	66.6	EL
TLINERWLLTTAK	185–197	3	573.6340	1	5.77	71.9	LL
LINERWLLTTAKNL	186–199	3	615.6573	1	6.20	70.1	LL
TPTLRLYVGKNQ	213–224	2	735.3677	2	6.35	72.4	LL
LYVGKN	218–223	2	347.1990	1	5.81	63.2	LF
GKNQLVEVE	221–229	2	508.2893	1	5.12	67.1	LL
KNQLVEVEK	222–230	2	543.8118	1	9.18	61.3	EL
KNQLVEVEKVVLHPDHSKV	222–240	3	760.0516	1	5.80	65.6	EF
LVEVEKVV	225–232	2	457.7771	1	6.19	64.2	EF
VEVEKV	226–231	2	351.7056	3	8.00	74.3	LF, EL, LL
VLHPDHSKV	232–240	2	516.3051	1	7.23	73.3	EF
VDIGLIK	240–246	2	379.2430	1	10.46	72.0	EL
IGLIK	242–246	1	543.3856	3	7.38	69.0	LF, LL
IGLIKLRQ	242–249	2	470.8007	3	9.99	75.9	LF, EL, LL
LIKLRQKVPVN	244–254	3	436.6078	1	6.26	73.1	LL
KVPVNDKVMPICLPS	250–264	2	820.4736	3	8.85	63.2	EL, LL
MPICLPSKDYV	258–268	2	633.3144	3	7.08	69.2	EL, LL
ICLPSK	260–265	1	660.3920	3	6.95	70.3	LF, LL
GRNENFNFTEHLKYVMLPVA	280–299	3	820.4142	1	5.45	64.0	LL
LKYVMLPVADQDKCVK	291–306	3	641.6717	1	5.82	68.0	EL
MLPVADQDKCVKHYEGVDAPKNKTAKS	295–321	3	1010.5347	4	7.60	71.5	EF, LF, EL, LL
LPVADQDK	296–303	2	443.2458	2	8.29	66.9	LF, LL
VDAPKNKTAKSPVGV	311–325	3	504.2912	1	5.37	61.5	LF
APKNKTAKSPVGVQPILN	313–330	4	466.2680	3	8.60	74.6	EF, LF, LL
VGLSKYQDDTCYGDAGSAFV	336–355	2	1077.0104	3	7.02	65.0	LF, EL, LL
LSKYQD	338–343	2	377.1974	1	5.60	66.9	LL
TWYAAGILSFDKSC	363–376	3	540.2625	3	11.46	72.3	LF, EL, LL
ILSFDKSC	369–376	2	485.2376	2	8.17	66.7	EL, LL
ILSFDKSCAVAEYGVY	369–384	3	607.9783	1	6.77	68.2	LL

### Effect of haptoglobin on embryonic development, quality and gene expression during in vitro culture

To determine the optimal concentration of haptoglobin in IVC media different concentrations were tested. Using 20 µg/mL of haptoglobin (H20), the development at 96 hpi (≥ 16-cell stage), and day 7 (blastocyst yield) was lethal (0.0%), while in control (Control) it was the expected results at 48 hpi: approximately 87.7 ± 0.6%; 96 hpi (≥ 16-cell stage): approximately 70.5 ± 0.3%; and days 7 and 8 (blastocyst yield): approximately 22.1 ± 0.6% − 27.6 ± 0.5%. For 10 µg/mL (H10) and 5 µg/mL (H5), the cleavage rate at 48 hpi was: 86.9 ± 0.7% and 88.2 ± 0.7%, at 96 hpi (range of ≥ 16-cell stage embryos: 71.4 ± 0.6% and 72.0 ± 0.5%) and days 7–8 (range of blastocyst yield: 19.2 ± 0.3% and 21.2 ± 0.2% and 27.3 ± 0.4% and 32.5 ± 0.4%, respectively) being higher to 5 µg/mL of haptoglobin concentration (Additional file 4).

Concerning the above, concentration of haptoglobin was decreased. Therefore, presumptive zygotes from four individual replicates were cultured in IVC medium supplemented with 2.5 µg/mL of haptogolobin (H2.5: *n* = 227) until day 8. The development at 48 hpi (86.6 ± 1.2%), 96 hpi (≥ 16-cell stage- 71.2 ± 0.7%) and day 7–8 (range of blastocyst yield: 21.4 ± 0.7%–27.6 ± 0.2%, respectively) being similar to control group (Additional file 4). Consequently, concentration of 5 µg/mL of haptoglobin were used in the following experiments.

**Fig. 3:**
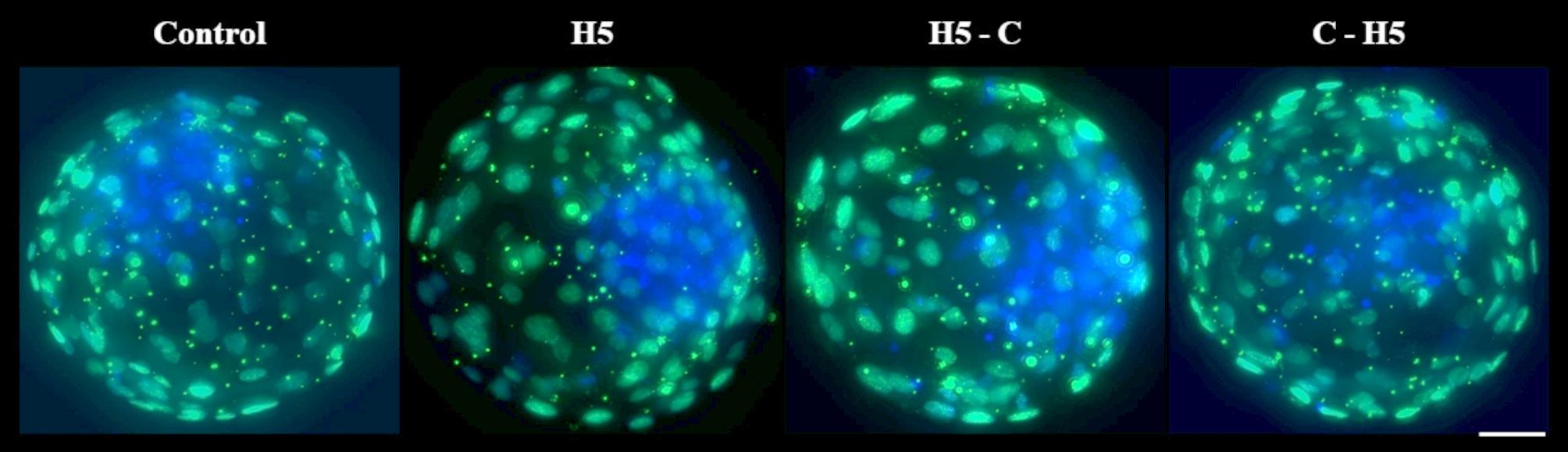
Representative fluorescent images of differential staining of bovine blastocysts cultured in the presence of haptoglobin. Control: presumptive zygotes cultured in the presence of IVC media. H5: presumptive zygotes cultured in IVC media supplemented with 5 µg/mL of haptoglobin; H5-C: from presumptive zygotes to 16-cell stage cultured in IVC media supplemented with 5 µg/mL of haptoglobin; and C-H5: embryos from 16-cell to blastocyst stage cultured in IVC media supplemented with 5 µg/mL of haptoglobin. Inner cell mass is shown in blue and trophectoderm cells are shown in green. Scale bar 25 μm

There were no significant differences in the proportion of embryos reaching the 16-cell stage at 96 hpi, with values ranging from 70.6 ± 0.4% to 71.7 ± 0.5%. Similarly, the proportion of embryos exhibiting delayed development (< 16 cells) remained comparable across groups, ranging from 15.7 ± 0.6% to 17.8 ± 0.6%. In contrast, blastocyst yield on days 7 and 8 was significantly higher (*P* < 0.001) in the H5 group (28.3 ± 0.4% and 32.8 ± 0.6%, respectively) and the C-H5 group (27.4 ± 0.6% and 32.2 ± 0.9%) compared to the Control group (23.0 ± 0.5% and 27.9 ± 0.6%) and the H5-C group (23.7 ± 0.6% and 28.3 ± 0.7%) (Table 2). Moreover, total cell, TE and ICM cells did not show differences in blastocysts cultured among different experimental groups (Table 3; Fig. 3).

**Table 2:** Cleavage rate and kinetics of development at 96 h post – insemination and cumulus blastocysts rates on days 7 and 8 after IVC with or without haptoglobin supplementation

		Total cleaved 48 hpi	Development rates at 96 hpi		Blastocysts	
	IVC *N*	*N* (%±s.e.m.)	< 16 cells *N* (%±s.e.m.)	≥ 16 cells *N* (%±s.e.m.)	Day 7 *N* (%±s.e.m.)	Day 8 *N* (%±s.e.m.)
Control	516	454 (88.2 ± 0.5)	91 (17.6 ± 0.6)	365 (70.6 ± 0.4)	106 (23.0 ± 0.5)^b^	128 (27.9 ± 0.6)^b^
H5	519	459 (88.4 ± 0.6)	82 (15.8 ± 0.7)	377 (71.5 ± 0.3)	132 (28.3 ± 0.4)^a^	153 (32.8 ± 0.6)^a^
H5-C	438	386 (88.4 ± 0.5)	78 (17.8 ± 0.6)	446 (70.6 ± 0.6)	90 (23.7 ± 0.6)^b^	108 (28.3 ± 0.7)^b^
C-H5	446	389 (87.2 ± 0.5)	70 (15.7 ± 0.6)	309 (71.7 ± 0.5)	108 (27.4 ± 0.6)^a^	127 (32.2 ± 0.9)^a^

Control: presumptive zygotes cultured in the presence of IVC media. H5: presumptive zygotes cultured in IVC media supplemented with 5 µg/mL of haptoglobin. H5-C: from presumptive zygotes to 16-cell stage cultured in IVC media supplemented with 5 µg/mL of haptoglobin. C- H5: embryos from 16-cell to blastocyst stage cultured in IVC media supplemented with 5 µg/mL of haptoglobin. Data are the mean ± s.e.m. Within columns, different superscript letters indicate significant difference (*P* < 0.001) between treatments

**Table 3:** Embryo quality assessment of bovine embryos after IVC with or without haptoglobin

	No. Blastocysts Processed	No. Total nuclei	No. ICM nuclei	No. TE nuclei	Ratio ICM/TE
Control	25	118.6 ± 1.1	41.0 ± 0.7	76.9 ± 0.9	0.5 ± 0.01
H5	20	117.1 ± 0.9	40.5 ± 0.7	76.6 ± 0.9	0.5 ± 0.01
H5-C	21	117.2 ± 0.9	41.5 ± 0.5	75.7 ± 0.7	0.5 ± 0.01
C-H5	21	117.7 ± 1.0	41.4 ± 0.8	76.2 ± 1.0	0.5 ± 0.01

Control: presumptive zygotes cultured in the presence of IVC media. H5: presumptive zygotes cultured in IVC media supplemented with 5 µg/mL of haptoglobin; H5-C: from presumptive zygotes to 16-cell stage cultured in IVC media supplemented with 5 µg/mL of haptoglobin; and C- H5: embryos from 16-cell to blastocyst stage cultured in IVC media supplemented with 5 µg/mL of haptoglobin. ICM: inner cell mass; TE: trophectoderm. Data are the mean ± s.e.m. Within columns, different superscript letters indicate significant difference (*P* ≤ 0.001) between treatments

**Fig. 4:**
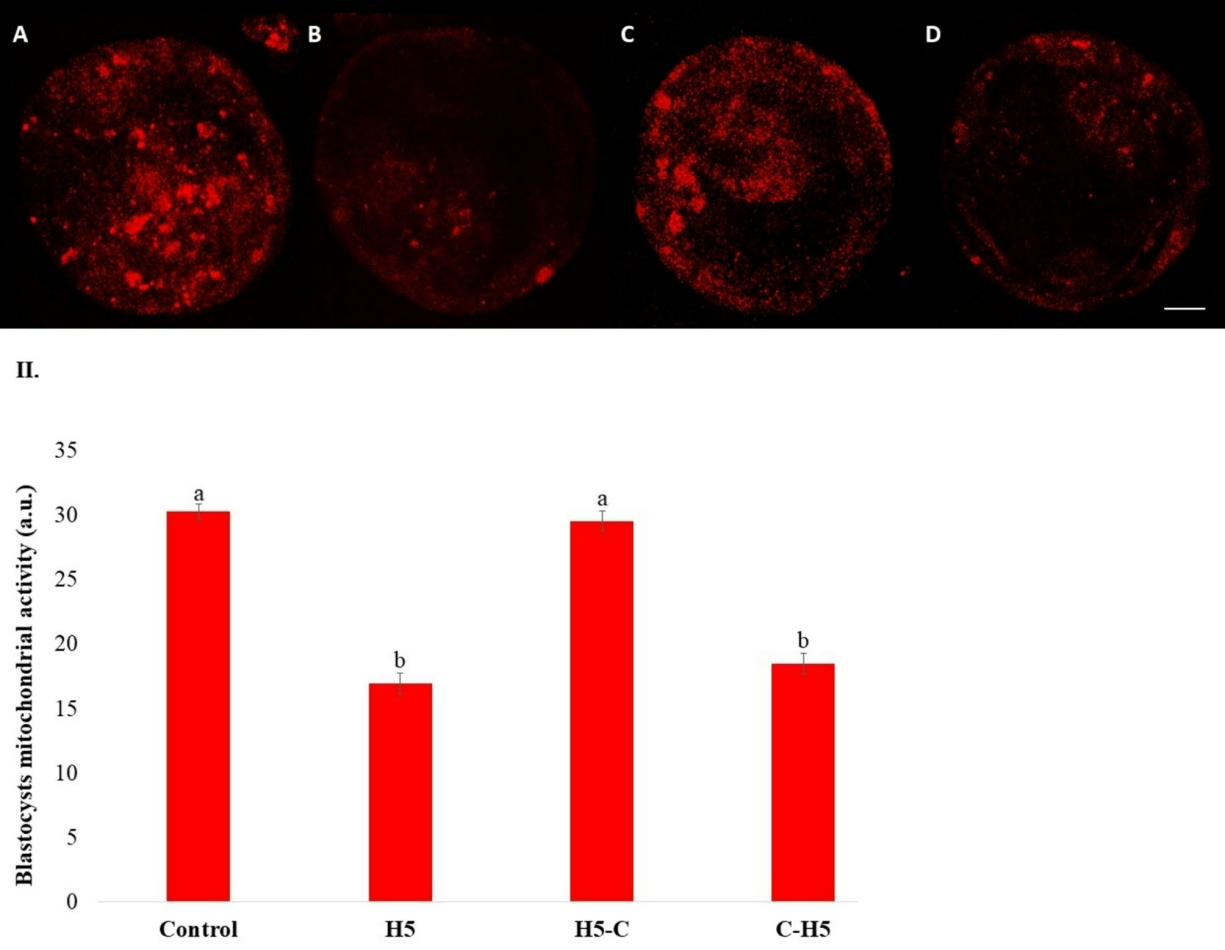
Mitochondrial fluorescent intensity in bovine blastocysts cultured in the presence of haptoglobin. **I).** Representative fluorescent images of mitochondria activity blastocysts cultured with haptoglobin. (**A**) Control: presumptive zygotes cultured in the presence of IVC media. (**B**) H5: presumptive zygotes cultured in IVC media supplemented with 5 µg/mL of haptoglobin; (**C**) H5-C: from presumptive zygotes to 16-cell stage cultured in IVC media supplemented with 5 µg/mL of haptoglobin; and (**D**) C- H5: embryos from 16-cell to blastocyst stage cultured in IVC media supplemented with 5 µg/mL of haptoglobin. Scale bar 25 μm. **II).** Quantification of mitochondrial fluorescence intensity on day 7 blastocysts cultured with haptoglobin. Data are the mean ± SEM. Values with different superscript letters differ significantly (*P* < 0.05)

**Fig. 5:**
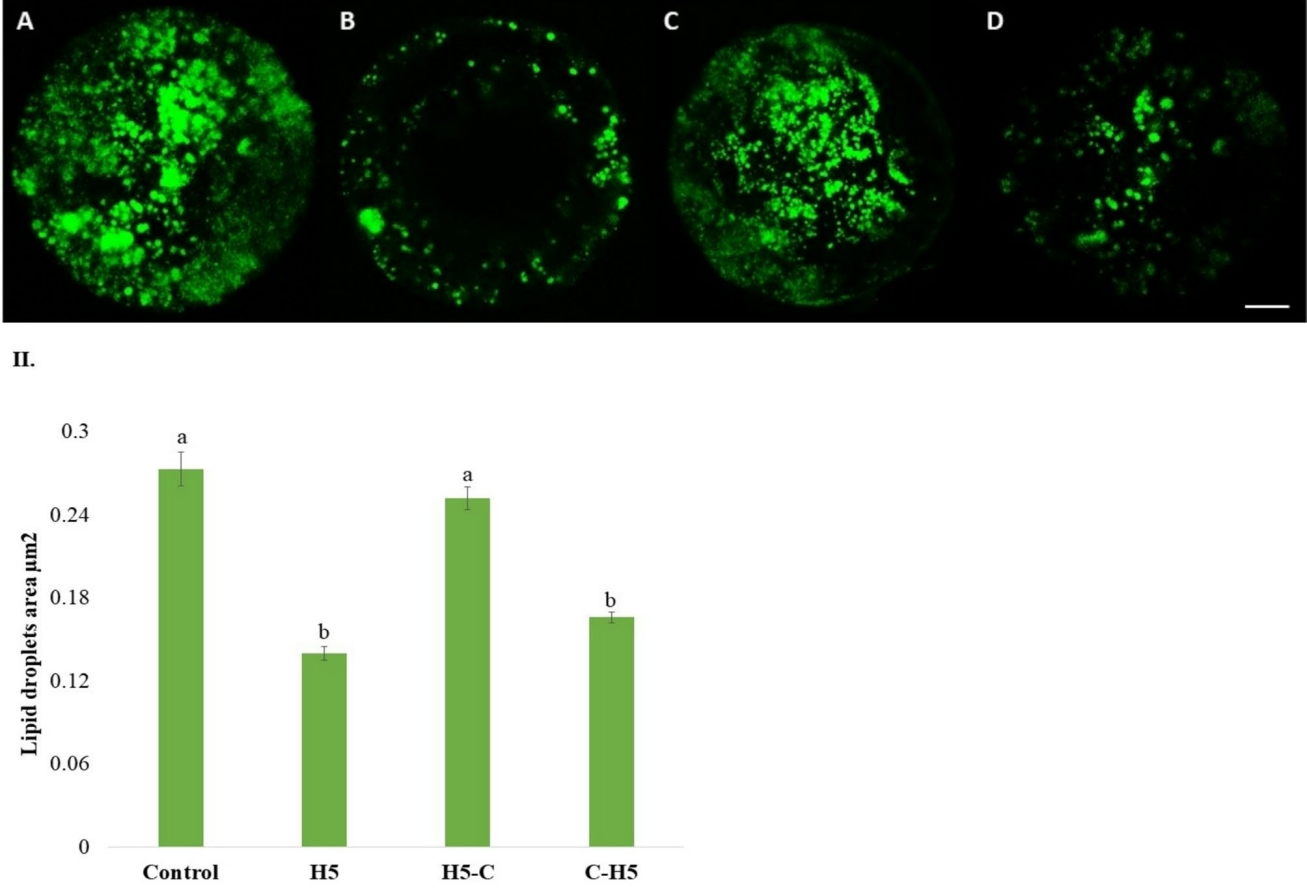
Lipid content in bovine blastocysts cultured in the presence of haptoglobin. **I)** Representative fluorescent images of lipid droplets in blastocysts cultured with haptoglobin. (**A**) Control: presumptive zygotes cultured in the presence of IVC media. (**B**) H5: presumptive zygotes cultured in IVC media supplemented with 5 µg/mL of haptoglobin; (**C**) H5-C: from presumptive zygotes to 16-cell stage cultured in IVC media supplemented with 5 µg/mL of haptoglobin; and (**D**) C-H5: embryos from 16-cell to blastocyst stage cultured in IVC media supplemented with 5 µg/mL of haptoglobin. Scale bar 25 μm. **II).** Quantification of lipid droplets on day 7 blastocysts cultured with haptoglobin. Data are the mean ± SEM. Values with different superscript letters differ significantly (*P* < 0.05)

Mitochondrial activity was significantly lower (*P* < 0.001) in blastocysts from groups treated with haptoglobin throughout the entire culture period (H5) or during days 1 to 4 (C-H5), compared to the Control and H5-C groups (Fig. 4). Additionally, lipid content, measured as the total area of lipid droplets, was significantly reduced (*P* < 0.001) in blastocysts from the H5 and C-H5 groups (Fig. 5).

**Fig. 6:**
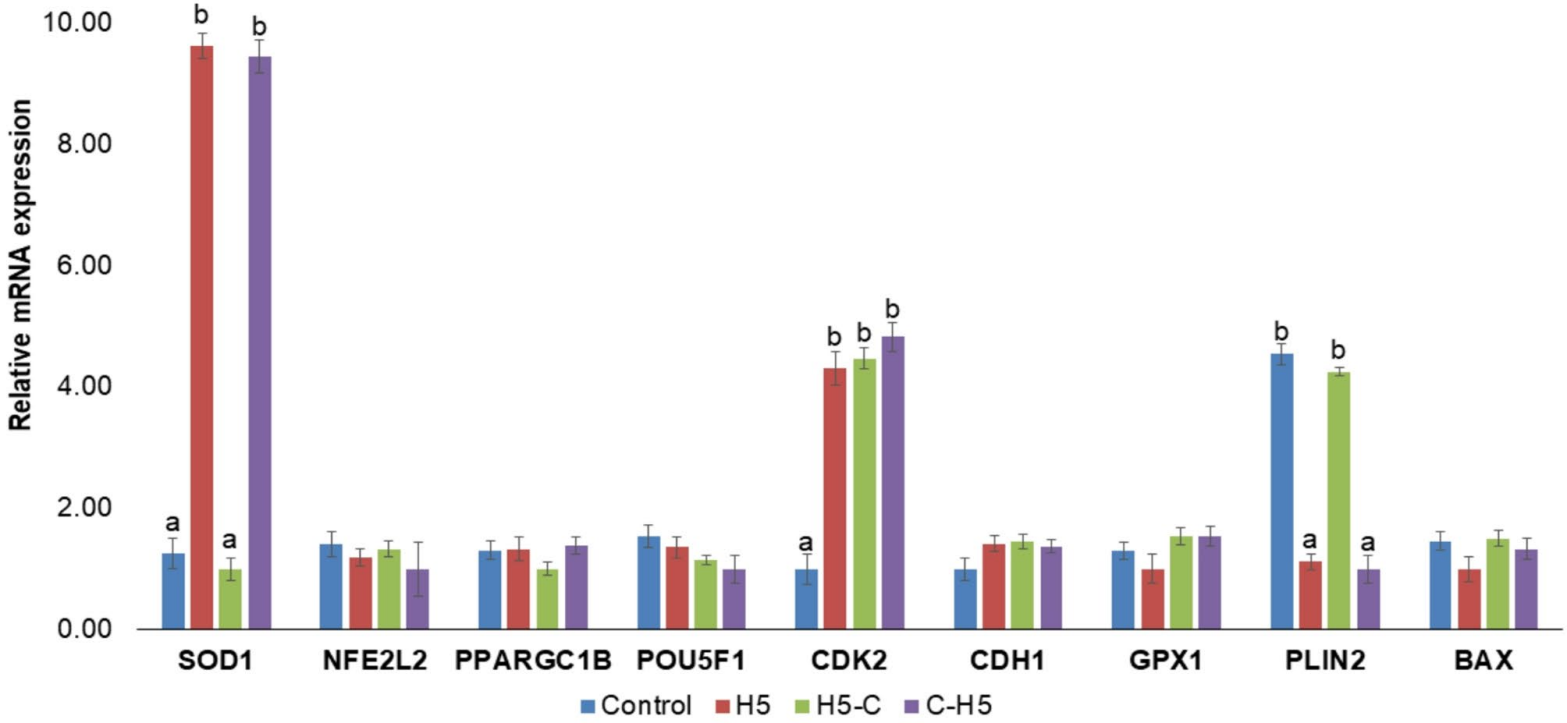
Relative mRNA transcript abundance of embryo development-related genes in in vitro produced embryos cultured with or without haptoglobin. Control: presumptive zygotes cultured in the presence of IVC media. H5: presumptive zygotes cultured in IVC media supplemented with 5 µg/mL of haptoglobin; H5-C: from presumptive zygotes to 16-cell stage cultured in IVC media supplemented with 5 µg/mL of haptoglobin; and C- H5: embryos from 16-cell to blastocyst stage cultured in IVC media supplemented with 5 µg/mL of haptoglobin. The relative abundance of the transcripts was normalized to H2AFZ and ACTB as housekeeping genes. Data are the mean ± s.e.m. Different letters indicate significant difference (*P* ≤ 0.05) between treatments

The expression level of *SOD1* transcripts was significantly increased in blastocysts from H5 and C-H5 groups compared to control and H5-C groups (*P* < 0.05). Conversely, the expression of *PLIN2* gene was significantly higher in the Control and H5-C groups compared to the H5 and C-H5 groups (*P* < 0.05). Additionally, *CDK2* expression was upregulated in all haptoglobin-treated groups compared to the control (*P* < 0.05). No significant differences were observed in the expression of *NFE2L2*, *PPARGC1B*, *POU5F1*, *CDH1* and *BAX* in the blastocysts among the groups (Fig. 6).

**Fig. 7:**
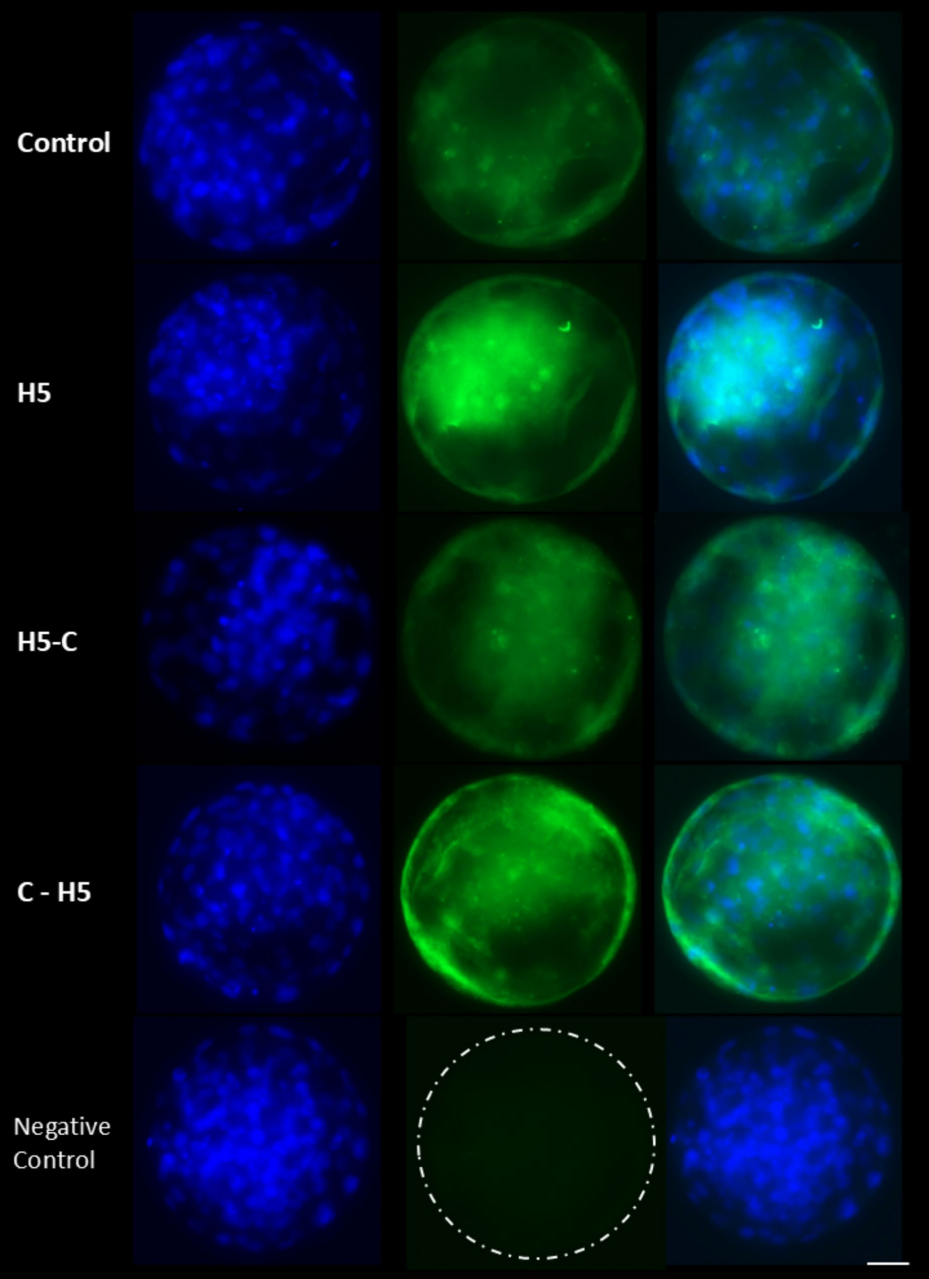
Representative images of immunofluorescence detection of SOD1 in in vitro produced bovine blastocysts cultured with haptoglobin. Control: presumptive zygotes cultured in the presence of IVC media. H5: presumptive zygotes cultured in IVC media supplemented with 5 µg/mL of haptoglobin; H5-C: from presumptive zygotes to 16-cell stage cultured in IVC media supplemented with 5 µg/mL of haptoglobin; and C- H5: embryos from 16-cell to blastocyst stage cultured in IVC media supplemented with 5 µg/mL of haptoglobin. Positive staining for SOD1 proteins shown in green. Cell nuclei were counterstained with Hoechst stain (blue). Images were captured on 63X objective. Scale bar 25 μm

**Fig. 8:**
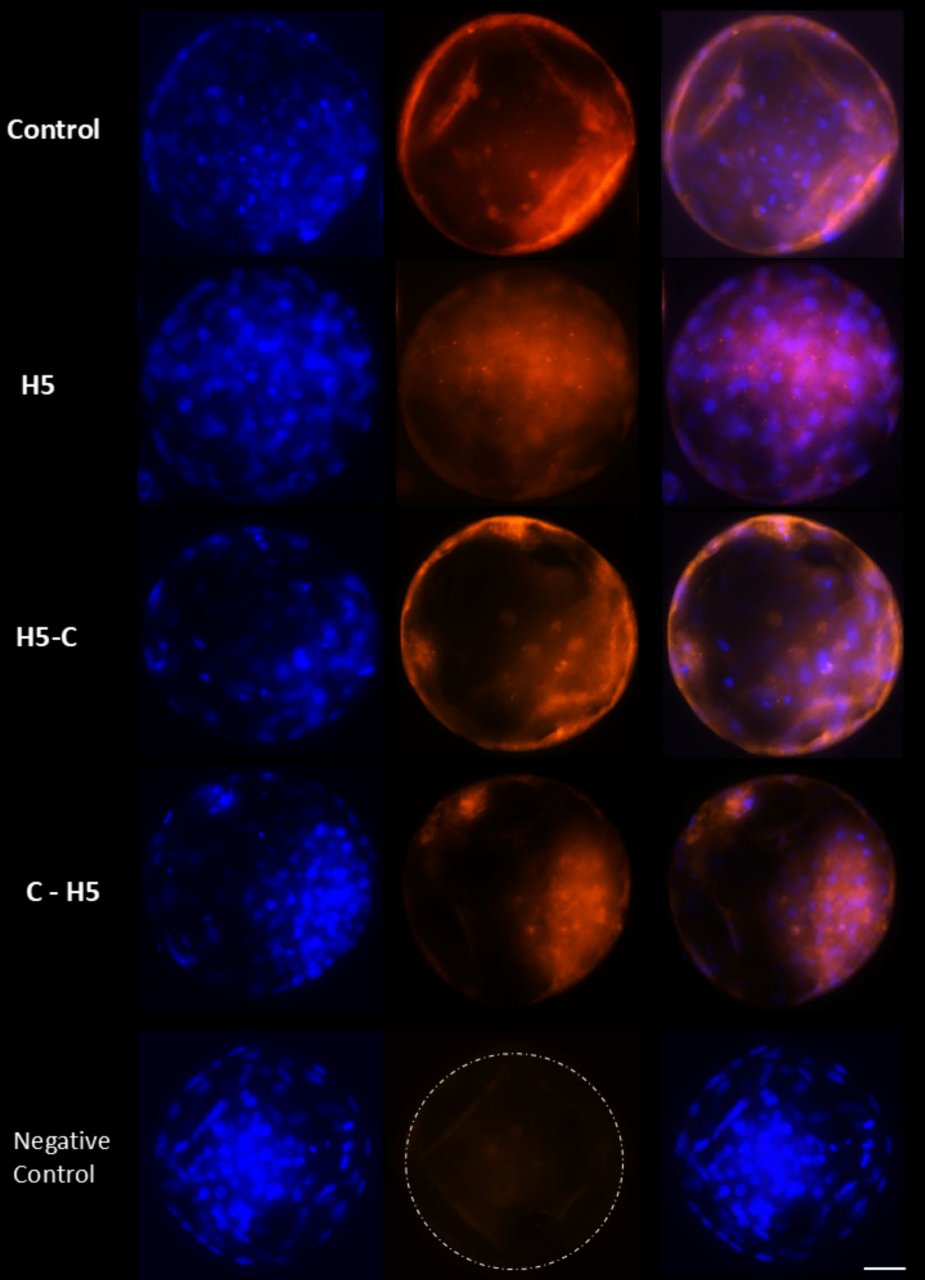
Representative images of immunofluorescence detection of PLIN2 in in vitro produced bovine blastocysts cultured with haptoglobin. Control: presumptive zygotes cultured in the presence of IVC media. H5: presumptive zygotes cultured in IVC media supplemented with 5 µg/mL of haptoglobin; H5-C: from presumptive zygotes to 16-cell stage cultured in IVC media supplemented with 5 µg/mL of haptoglobin; and C- H5: embryos from 16-cell to blastocyst stage cultured in IVC media supplemented with 5 µg/mL of haptoglobin. Positive staining for PLIN2 proteins shown in red. Cell nuclei were counterstained with Hoechst stain (blue). Images were captured on 63X objective. Scale bar 25 μm

Immunofluorescence analysis confirmed the presence of immunoreactive proteins SOD1 and PLIN2 in bovine blastocysts. Both proteins were predominantly localized in the cytoplasm of embryos cultured with 5 µg/mL of haptoglobin (H5) during day 4 to day 7: from 16-cell to blastocyst stage (representing haptoglobin effect in the uterus; C-H5), suggesting constant stimulation of these proteins during the preimplantation period. The fluorescence intensity of SOD1 was higher in blastocysts from the H5 and C-H5 groups (Fig. 7), whereas PLIN2 levels were weaker in these groups compared to other conditions (Fig. 8).

## Discussion

In the last decades, IVP of cattle embryos has considerably increased. However, only 30 to 40% of embryos develop to blastocyst stage, and approximately 50% of transferred embryos establish a pregnancy [57]. This highlights the necessity to enhance the efficacy of current IVP which includes IVM, IVF, and IVC systems. The presence of haptoglobin in the female reproductive fluids of several species [16, 33, 34, 44–48, 54] suggests that this protein may play a role in modulating gamete interaction and early embryo development. Our findings suggest that haptoglobin is secreted by epithelial cells to the uterus lumen, and that its expression levels fluctuate throughout the oestrous cycle in bovine species. In our initial approach, the expression of haptoglobin in the bovine uterus was analysed. RT-PCR demonstrated that haptoglobin was present in bovine uterus (body and horns). Moreover, RT-qPCR and immunohistochemistry demonstrated its presence in the uterus throughout the oestrous cycle showing the highest expression in the LL phase and the lowest in the EF phase. Previous studies have also detected haptoglobin expression in bovine endometrium in absence of inflammation [39]; however, no significant differences were found through the oestrus cycle [39]. On the other hand, we previously reported similar findings in porcine species, where haptoglobin expression was detected through the oestrous cycle, with the expression increasing in the EL phase, reaching a maximum expression in the LL phase [15]. In rabbits, haptoglobin expression in the endometrium was detectable from day 1 of pregnancy, increasing until day 5 and reaching a maximum on day 6 (the last day investigated) [34]. On the other hand, in women, haptoglobin expression in the endometrium was higher during the luteal phase than the proliferative phase [37]. This finding is consistent with our own results, which showed that haptoglobin levels were higher in the LL phase than in the LF phase. In cattle, the LL phase lasts approximately 18 days and includes the phase of luteal body formation, and haptoglobin expression appears to coincide with the increased presence of progesterone (secreted by the corpus luteum) and the reduction in oestrogen levels. This is consistent with the results observed in several studies and seems to confirm the relationship between haptoglobin and preimplantation embryo development, which occurs in the oviduct and uterus during the luteal phase [15, 58].

Previous studies have demonstrated the presence of haptoglobin in the bovine OF by means of Western blot and proteomic analysis [32, 43]. In this study, the presence of haptoglobin in the UF was studied by proteomic analysis in different oestrous stages (EF, LF, EL, LL). Our results indicate the presence of haptoglobin in uterus in all the oestrus phases, which seems to confirm the presence of haptoglobin in the microenvironment surrounding the pre-implanted embryo and led us to perform functional analyses using this protein. Moreover, our experiments showed that the incorporation of this protein into the IVC media enhances the efficacy of IVP bovine embryos. A significant effect was observed when haptoglobin is present during all the embryo culture (H5 group) and in C-H5 group that represents haptoglobin effect in the uterus compared to control group. These results indicate that haptoglobin protein exerts its effect when it is present in the uterus, as previously demonstrated for porcine species [16]. Moreover, the quality of embryos is higher when the haptoglobin is present in the whole IVC (H5) or in the last phase of in vitro development (C-H5). Thus, mitochondrial activity was lower (*P* < 0.001) in blastocysts from H5 and C-H5 groups (corresponding with presence of haptoglobin in uterus), compared with control and H5-C. Some studies have indicated that alterations in mitochondrial activity could potentially influence the development of energetic metabolism in the embryo, specifically with regard to the availability of amino acids, glucose, lipids, and DNA methylation [59, 60]. The lipid content is lower in the same groups (H5 and C-H5), which indicates that the blastocysts recovered in these groups are of a higher quality. Recent studies described that the cells of embryos derived in vivo contained cytoplasmic lipid droplets but were not abundant [61, 62]. Abe et al. [63]. showed that the cells of low-quality embryos contained larger numbers of lipid droplets than the high-quality embryos. The reason of lipid accumulation in low quality bovine embryos is unclear. More recent studies demonstrated that the morulae and blastocysts developed in a medium supplemented with calf serum were characterized by the accumulation of a large number of cytoplasmic lipid droplets, while those cultured in the serum-free medium had fewer lipid droplets, suggesting that the presence of calf serum in embryo culture medium is the specific cause for the accumulation of cytoplasmic lipid droplets [57, 64].

All these results might indicate that haptoglobin plays a role in embryo development after the first divisions. In rabbits, haptoglobin is present in extra-embryonic matrix and blastocyst fluid, although no expression has been detected in the embryo, which suggests that the embryo is able to uptake haptoglobin from the surrounding fluids, as the embryo passes through the maternal reproductive tract [34]. In fact, the uptake of proteins by embryos differs between the early embryo and blastocyst stages [65–67], which indicates that embryos can use components depending on the developmental stage or surrounding conditions. Bovine embryo genome activation occurs at the eight- to 16-cell stage [68–70]. In light of the aforementioned results, it seems plausible to suggest that the intake of haptoglobin from this stage may have a beneficial effect on the embryo development and implantation. However, no differences were detected between the H5 and C-H5 groups, suggesting that haptoglobin may not be indispensable during the embryonic transit through the oviduct.

On the other hand, haptoglobin is able to change the gene expression of the embryos. We analysed the expression of candidate genes for cell proliferation and embryo quality in blastocysts, such as *POU5F1*, *CDK2*, *CHD1*, *PPARGC1B*, *SOD1*, *PLIN2*, *GPX1*, *NFE2L2*, *BAX* [49–53]. Only three genes showed significant differences: *SOD1*, *PLIN2* and *CDK2*.

*SOD1* is an antioxidant enzyme expressed by cells to combat oxidative stress, being capable of dismutating the superoxide radicals into O_2_ and H_2_O_2_ [71, 72]. *SOD1* is upregulated in blastocysts of H5 and C-H5 groups compared with controls, this result could indicate that these embryos have a higher antioxidative ability. However, this gene was previously studied in sheep embryos, and it was unaffected after the embryo culture with L-carnitine [73].

*PLIN2* is a known marker of cellular lipid accumulation [74] and directly interacts with lipids on the droplet surface [75, 76]. *PLIN2* is downregulated in the groups where haptoglobin is present throughout the culture (H5) and in the group that simulates its presence in the uterus (C-H5). These results are in accordance with the fluorescent images of lipid droplets in blastocysts and the quantification, showing a reduction of the lipid content in the same stages. In vitro-produced embryos from domestic animals frequently exhibit higher quantity of lipid drops when compared with their in vivo-produced counterparts [77–79]. Reduction of post thaw viability is associated with the abnormal quantity of lipid drops. Moreover, it has been suggested that excess accumulation of cytoplasmic lipids may affect various physiological characteristics of embryos, being more sensitive to cryopreservation procedures [64, 80, 81], because they contribute to the occurrence of cryofractures during the freezing process [82–84]. Our results could indicate that the lipid droplets are reduced in these groups being an advantage for the embryo freezing. This is of particular importance in the bovine industry, as in this species, embryos are frozen and vitrified for transport and subsequent transfer.

*CDK2* is essential for cell cycle progression and serves as a key kinase regulating AKT phosphorylation. Additionally, it plays a role in embryo genome activation [85]. In our study, *CDK2* mRNA expression was upregulated in blastocysts cultured with haptoglobin. This finding aligns with previous data from bovine embryos, which reported transcriptional changes in cell cycle-related genes and an increased *CDK2* expression during the blastocyst stage [86].

## Conclusions

The observations presented herein demonstrate that haptoglobin is secreted by the epithelium lining the uterine lumen and glandular cells during different phases of the oestrous cycle of the cow. These findings suggest that it plays an important role in early embryo development. The use of haptoglobin in culture media could have the potential to replicate the properties of physiological fluids, thereby enhancing the efficiency of IVP.

## Supplementary Information

Below is the link to the electronic supplementary material.

**Additional file 1.** Primers for RT-PCR and RT-qPCR amplification of haptoglobin and ACTB genes.

**Additional file 2**. Primers used for blastocysts gene expression analysis by RT-qPCR.

**Additional file 3.** Haptoglobin protein sequence (NP_001035560). Peptides detected by HPLC-ESI-MS/MS appear underlined.

**Additional file 4.** Preliminary experiment: Cleavage rate and kinetics of development at 96 h post – insemination and cumulus blastocysts rates on days 7 and 8 after IVC with or without haptoglobin supplementation.

## Data Availability

The data used to support the results of the current study are available from the corresponding authors upon request.

## Abbreviations

ACTB: β-actin
ARTs: Assisted reproductive technologies
BAX: BCL2 associated X, apoptosis regulator
BSA: Bovine serum albumin
CDK2: Cyclin-dependent Kinase 2
CHD1: Chromodomain Helicase DNA Binding Protein 1
COCs: Cumulus-oocyte complexes
dpi: Days post-insemination
EC: Embryo culture
EF: Early follicular
EL: Early luteal
FCS: Fetal calf serum
GPX1: Glutathione Peroxidase 1
H: Haptoglobin
HK: Housekeeping
hpi: Hours post-insemination
ICM: Inner cell mass
IVC: In vitro culture
IVF: In vitro fertilization
IVP: In vitro production
EL: Early luteal
LL: Late luteal
MMLV: Moloney murine leukaemia virus
NFE2L2: Nuclear Factor Erythroid 2-Related Factor 2
OF: Oviductal fluid
PCR: Polymerase chain reaction
PLIN2: Perilipin 2
PBS: Phosphate-buffered saline
PF: Paraformaldehyde
POU5F1: POU Domain, Class 5, Transcription Factor 1
PPARGC1B: PPARG Coactivator 1 Beta
PVP: Polyvinylpyrrolidone
RT: Reverse transcription
RT-PCR: Reverse transcription polymerase chain reaction
RT-qPCR: Reverse transcription quantitative real-time polymerase chain reaction
SOD1: Superoxide Dismutase 1
SWM: Semen wash media
TE: Trophectoderm
UF: Uterine fluid
WM: Wash media
