# Supplementary material for: Haptoglobin as a supplement in in vitro embryo culture: a tool for improving bovine embryo development and quality
Source: Biol Res. 2025 Aug 20;58:58. doi: 10.1186/s40659-025-00635-0 (PMC12366215; doi:10.1186/s40659-025-00635-0)
Supplement: Supplementary file 1 — Additional file 1. Primers for RT-PCR and RT-qPCR amplification of haptoglobin and ACTB genes. [file 40659_2025_635_MOESM1_ESM.docx]

**Additional file 1.** Primers for RT-PCR and RT-qPCR amplification of haptoglobin and ACTB genes

| **Gene** | **bp (f/r)** | **Sequence** | **Tm (ºC)** | **Genbank/Ensembl accession number** |
| --- | --- | --- | --- | --- |
| RT-PCR primers | | | | |
| **Haptoglobin** | 19 (f) | GGGTCGTGTGGGTTATGTG | 60 | NM_001040470 |
| **Haptoglobin** | 19 (r) | CGACGCAGAAGGTGTTCTC | 60 | NM_001040470 |
| **ACTB** | 19 (f) | CTTCCAGCCTTCCTTCCTG | 60 | NM_173979 |
| **ACTB** | 19 (r) | CGGACTCATCGTACTCCTG | 60 | NM_173979 |
| Real time RT-PCR primers | | | | |
| **Haptoglobin** | 20 (f) | GTTCGCTATCAGTGCAAACC | 60 | ENSBTAG00000006354 |
| **Haptoglobin** | 20 (r) | CCTCACATTCAGGGAGTTTC | 60 | ENSBTAG00000006354 |
| **ACTB** | 19 (f) | GAGAAGCTCTGCTACGTGG | 60 | ENSBTAG00000026199 |
| **ACTB** | 19 (r) | GTTTCGTGAATGCCGCAGG | 60 | ENSBTAG00000026199 |
