# Supplementary material for: Haptoglobin as a supplement in in vitro embryo culture: a tool for improving bovine embryo development and quality
Source: Biol Res. 2025 Aug 20;58:58. doi: 10.1186/s40659-025-00635-0 (PMC12366215; doi:10.1186/s40659-025-00635-0)
Supplement: Supplementary file 2 — Additional file 2. Primers used for blastocysts gene expression analysis by RT-qPCR. [file 40659_2025_635_MOESM2_ESM.docx]

**Additional file 2**. Primers used for blastocysts gene expression analysis by RT-qPCR.

| **Gene** | **Gene name** | **Primer sequence (5’- 3’)** | **Fragment size (bp)** | **GenBank accession number** |
| --- | --- | --- | --- | --- |
| ACTB | Actin, beta | F: GAGAAGCTCTGCTACGTCG  R: CCAGACAGCACCGTGTTGG | 264 | AF191490.1 |
| BAX | BCL2- Associated X Protein | F: TGGAGCAGGTGCCTCAGGA  R: ATCTCGAAGGAAGTCCAGCGTC | 300 | NM_001166486.1 |
| CDH1 | Cadherin 1 | F: GATTGCAAGTTCCCGCCATC  R: ACATTGTCCCGGGTGTCATC | 144 | NM_001002763 |
| CDK2 | Cyclin Dependent Kinase 2 | F: TCTTTGCTGAGATGGTGACCC  R: GTTAGGGTCGTAGTGCAGCAT | 242 | NM_001014934.1 |
| GPX1 | Glutathione Peroxidase 1 | F: GCAACCAGTTTGGGCATCA  R: CTCGCACTTTTCGAAGAGCATA | 116 | NM_174076.3 |
| H2AFZ | H2A histone family, member Z | F: AGGACGACTAGCCATGGACGTGTG  R: CCACCACCAGCAATTGTAGCCTTG | 212 | NM_016750 |
| NFE2L2 | Nuclear Factor Erythroid 2-Like 2 | F: CAGGACATTGAGCAAGTTTGG  R: GTGGAAAGGATGCTGTTGAAG | 234 | NM_001011678.2 |
| PLIN2 | (perilipin 2) | F: ACAACACACCCCTCAACTGG  R: CTGCCTGCCTACTTCAGACC | 2111 | NM_173980.2 |
| PPARGC1A | PPARG coactivator 1 alpha | F: AAAAGCCACAAAGACGTCCG  R: TCTGCTGCTGTTCCGGTTCT | 111 | NM_177945 |
| POU5F1 | POU domain, class 5, transcription factor 1(Oct3/4) | F: CGAGTATCGAGAACCGAGTG  R: CAGGGTTCTCTCCCTAGCTC | 440 | NM_174580.1 |
| SOD1 | Superoxide dismutase 1, soluble | F: CCCATGAAGCCTTTCTAATCC  R: TTCAGAGGGGCTACTATTTCC | 309 | NM_174615.2 |
