# Supplementary material for: Haptoglobin as a supplement in in vitro embryo culture: a tool for improving bovine embryo development and quality
Source: Biol Res. 2025 Aug 20;58:58. doi: 10.1186/s40659-025-00635-0 (PMC12366215; doi:10.1186/s40659-025-00635-0)
Supplement: Supplementary file 3 — Additional file 3. Haptoglobin protein sequence (NP_001035560). Peptides detected by HPLC-ESI-MS/MS appear underlined. [file 40659_2025_635_MOESM3_ESM.docx]

**Additional file 3.** Haptoglobin protein sequence (NP_001035560). Peptides detected by HPLC-ESI-MS/MS appear underlined.

1 MSALQAVVTL LLCGQLLAVE TGSEATADSC PKAPEIANSH VEYSVRYQCD KYYKLHAGNG

61 VYTFNNKQWI NKDIGQQLPE CEEDDSCPEP PKIENGYVEY LVRYQCKPYY TLRTCGDGVY

121 TFNSKKQWIN KNIGQKLPEC EAVCGKPKHP VDQVQRIIGG SLDAKGSFPW QAKMVSQHNL

181 ISGATLINER WLLTTAKNLY LGHSSDKKAK DITPTLRLYV GKNQLVEVEK VVLHPDHSKV

241 DIGLIKLRQK VPVNDKVMPI CLPSKDYVKV GRVGYVSGWG RNENFNFTEH LKYVMLPVAD

301 QDKCVKHYEG VDAPKNKTAK SPVGVQPILN ENTFCVGLSK YQDDTCYGDA GSAFVVHDKE

361 DDTWYAAGIL SFDKSCAVAE YGVYVKVTSI LDWVRKTIAN N
