# Supplementary material for: Haptoglobin as a supplement in in vitro embryo culture: a tool for improving bovine embryo development and quality
Source: Biol Res. 2025 Aug 20;58:58. doi: 10.1186/s40659-025-00635-0 (PMC12366215; doi:10.1186/s40659-025-00635-0)
Supplement: Supplementary file 4 — Additional file 4. Preliminary experiment: Cleavage rate and kinetics of development at 96 h post – insemination and cumulus blastocysts rates on days 7 and 8 after IVC with or without haptoglobin supplementation. [file 40659_2025_635_MOESM4_ESM.docx]

**Additional file 4.** Preliminary experiment: Cleavage rate and kinetics of development at 96 h post – insemination and cumulus blastocysts rates on Days 7 and 8 after IVC with or without haptoglobin supplementation.

|  |  | **Total cleaved**  **48 hpi** | **Development rates at 96 hpi** | | **Blastocysts** | |
| --- | --- | --- | --- | --- | --- | --- |
|  | **IVC**  **N** | **N**  **(%±s.e.m.)** | **< 16 cells**  **N**  **(%±s.e.m.)** | **≥ 16 cells**  **N**  **(%±s.e.m.)** | **Day 7**  **N**  **(%±s.e.m.)** | **Day 8**  **N**  **(%±s.e.m.)** |
| **Control** | 348 | 305  (87.7±0.6)^a^ | 60  (17.1±0.8)^b^ | 245  (70.5±0.3)^b^ | 77  (22.1±0.6)^b^ | 96  (27.6±0.5)^b^ |
| **2,5 µg** | 227 | 193  (86.6±1.2)^a^ | 36  (16.0±0.6)^b^ | 161  (71.2±0.7)^c^ | 48  (21.4±0.7)^b^ | 62  (27.6±0.2)^b^ |
| **5 µg** | 348 | 307  (88.2±0.7)^a^ | 56  (16.1±0.8)^b^ | 251  (72.0±0.5)^a^ | **95**  **(27.3±0.4)^a^** | **113**  **(32.5±0.4)^a^** |
| **10 µg** | 292 | 254  (86.9±0.7)^a^ | 46  (15.5±0.9)^b^ | 208  (71.4±0.6)^c^ | 56  (19.2±0.3)^c^ | 62  (21.2±0.2)^c^ |
| **20 µg** | 126 | 98  (77.3±2.2)^b^ | 98  (77.3±0.9)^a^ | 0 | 0 | 0 |

Data are the mean ± s.e.m. Within columns, different superscript letters indicate significant difference (P<0.001) between treatments.
